# Association between the domestic use of solid cooking fuel and increased prevalence of depression and cognitive impairment in a big developing country: A large-scale population-based study

**DOI:** 10.3389/fpubh.2022.1038573

**Published:** 2022-11-24

**Authors:** Yuming Jin, Xianghong Zhou, Linghui Deng, Xingyu Xiong, Yifan Li, Qiang Wei, Birong Dong, Shi Qiu

**Affiliations:** ^1^Department of Urology, National Clinical Research Center for Geriatrics, Institute of Urology, West China Hospital, Sichuan University, Chengdu, China; ^2^National Clinical Research Center of Geriatrics, The Center of Gerontology and Geriatrics, West China Hospital, Sichuan University, Chengdu, China; ^3^Institute of Oncology Research (IOR) and Oncology Institute of Southern Switzerland (IOSI), Bellinzona, Switzerland

**Keywords:** indoor air pollution, solid fuel, depression, older adults, cognitive impairment

## Abstract

**Background:**

Previous studies have suggested that air pollution affects physiological and psychological health. Using solid fuel at home is a significant source of indoor air pollution. The associations between solid fuel use and depressive symptoms and cognitive health were unclear among older adults from low- and middle-income countries (LMICs).

**Methods:**

To evaluate the association of solid fuel use with depressive symptoms and cognitive health among older adults, we obtained data from the Longitudinal Aging Study in India (LASI) and excluded subjects younger than 60 years and without critical data (solid fuel use, depressive symptoms, and cognitive health). The 10-item Center for Epidemiologic Studies Depression Scale (CES-D-10) was used to assess depressive symptoms, with more than ten indicative of depression. Cognitive health was assessed using measures from the Health and Retirement Study (HRS), and subjects with the lowest 10th percentile were considered to have cognitive impairment. The participants' responses defined solid fuel use. Multivariable logistic regression, linear regression, subgroup analysis, and interaction tests were performed to appraise the relationship between solid fuel use and depression and cognitive impairment.

**Results:**

A total of 29,789 participants over 60 years old were involved in this study. Almost half of the participants (47.5%) reported using solid fuel for home cooking. Compared with clean fuel use, solid fuel use was related to an increased prevalence of depression [odds ratio (OR) 1.09, 95% CI 1.03–1.16] and higher CES-D-10 scores (β 0.23, 95% CI 0.12–0.35) after fully adjusted covariables. Using solid fuel was also related to a higher risk of cognitive impairment (OR 1.21, 95% CI 1.11–1.32) and a lower cognitive score (β −0.63, 95% CI −0.79 to −0.47) compared with those who used clean fuel. In the subgroup analysis, the prevalence of depression increased in females and non-smokers. The association of solid fuel use with depression and cognitive impairment exists in subgroups of BMI, economic status, caste, living area, education, and drinking.

**Conclusions:**

The use of solid fuel at home was associated with an increased prevalence of depression and cognitive impairment among older adults in India.

## Introduction

Older adults have received extensive attention for the decline in physical function and lifestyle changes in recent decades. Older adults suffer a higher risk of chronic diseases due to the influence of long-term unhealthy lifestyles and dysfunction (1, 2). As people get older, they are more likely to suffer from cognitive impairment owing to cerebral atrophy, which harms their quality of life (3–5). Loneliness, functional disabilities, and chronic diseases are commonly significant risk factors for depression in older adults (6–8). Depression is the most common mental disorder in older adults, and the prevalence is higher in older adults than in young and middle-aged adults (8–10). The economic costs of depression and cognitive decline in the elderly are substantial, and they will rise as the severity of their symptoms worsens due to the higher costs associated with treating their illness (11, 12). Therefore, it is vital to prevent depression and cognitive impairment. Social and environmental factors and lifestyle are also positive indicators of depression (13, 14). As reported previously, air pollution is associated with depression and cognitive impairment with dose response (15, 16).

Over the years, air pollution has been recognized as a significant threat to public health, especially in low- and middle-income countries (LMICs) (17). Previous studies have shed light on the associations between air pollution and chronic disease, as well as the fact that air pollution increases the risk of unsatisfactory healthy status (18–20). Similarly, there is more evidence that air pollution is related to the prevalence of mental disorders and is correlated with the aggravation of depressive symptoms and cognitive impairment (21, 22). Household solid fuel produces particulate matter (PM), polycyclic aromatic hydrocarbons (PAHs), nitrogen dioxide (NO_2)_, and carbon monoxide (CO), which are primary sources of indoor pollution and can have detrimental ramifications (23–26). Evidence from previous studies has suggested that oxide nanoparticles enter the central nervous system through the alveolar epithelium; PMs reduce the cognitive learning abilities of rats (27, 28). It is worth noting that indoor air pollution was linked to depressive symptoms and cognitive impairment among elderly adults (29, 30). It is unclear whether indoor pollution is simultaneously related to depression and cognitive impairment.

In India, with the largest elderly population in the world, there will be 316 million people aged 60 years and above in 2050 (31). Approximately 9 and 13.5% of older adults live with depression and cognitive impairment in India (32, 33). More homes continue to use solid fuels because of their low cost and the lack of predictability in their revenue; only 22.5% of homes use clean fuels, and only 10% of rural homes use clean fuels (34, 35). Hence, it is essential to determine how indoor solid fuel use affects human health. Due to the widespread use of solid fuel in India, it makes sense to focus on the health outcomes of solid fuel use based on its population. A study from India suggested that solid fuel use for cooking was a risk factor for depression among premenopausal females (36). However, solid fuels have an opaque association with depression and cognitive impairment in older Indian adults. Using data from the Longitudinal Aging Study in India (LASI), the present study aimed to evaluate the association between the use of solid fuels with depression and cognitive impairment.

## Methods

### Participants and design of the study

**Figure 1:**
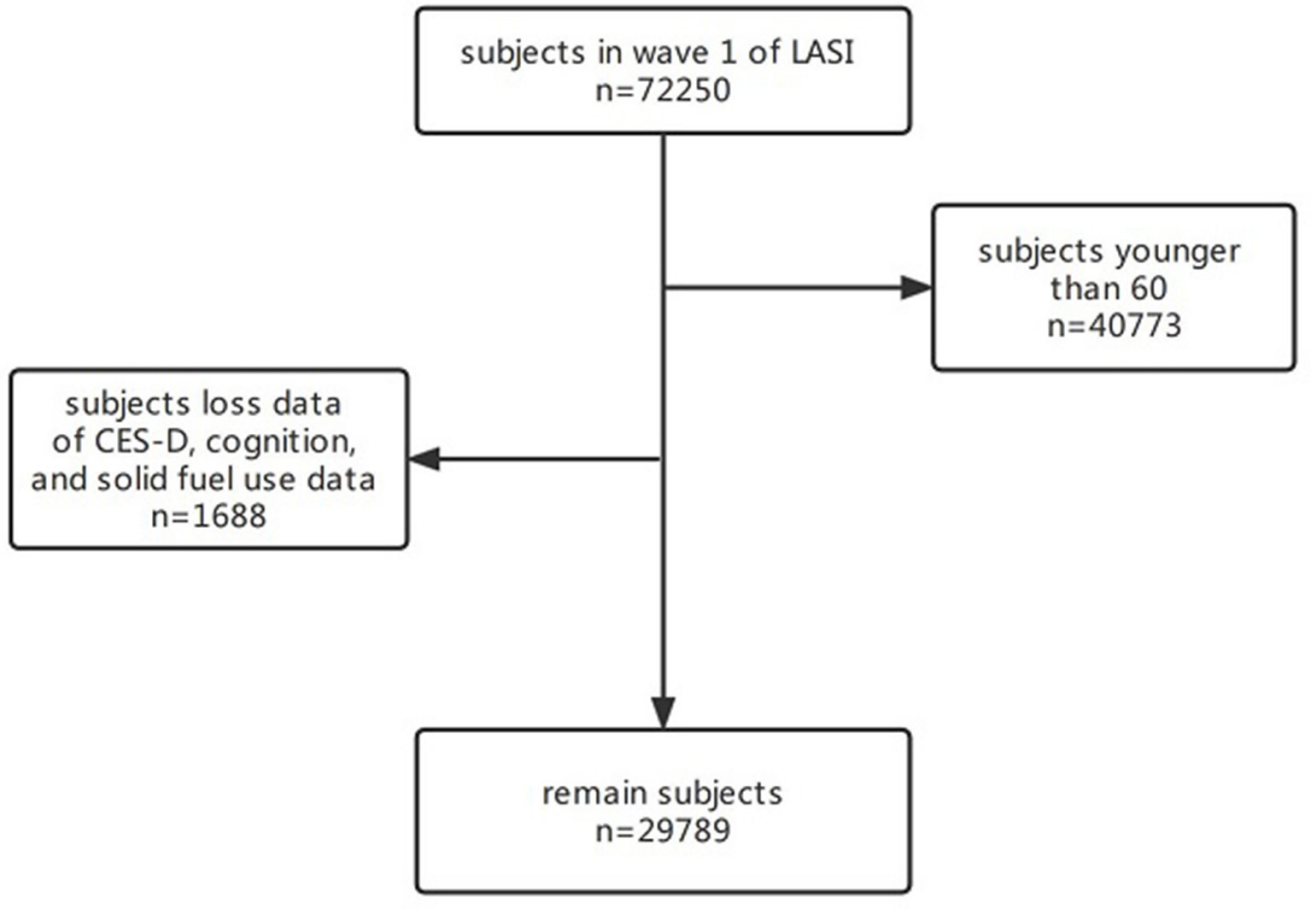
Inclusion and exclusion of the study population.

We performed a cross-sectional study to evaluate the association between solid fuel use with depression and cognitive impairment. Data for the present study came from the Longitudinal Aging Study in India (LASI). Health, economic, and social data from 72,250 adults aged 45 and up across all Indian states and union territories, with the exception of Sikkim, were collected over the course of 2 years (2017 and 2018) (37). LASI aimed to provide data regarding demography, financial status, self-reported health information, family, etc. In this study, we used the data from the first wave of the LASI. Variables of household fuel information and score of the 10-item Center for Epidemiologic Studies Depression Scale (CES-D-10) and the cognitive score of method from the Health and Retirement Study (HRS) were analyzed in the present study. We excluded those under 60 years old, those who lost data on household fuel details and CES-D-10 scores, and those without information on their cognition. Finally, 29,789 participants were included in this study. The inclusion and exclusion criteria of the study population are shown in Figure 1.

### Definition of solid fuel use

In the LASI study, subjects were required to answer the type of cooking fuel. Participants who reported using clean fuels such as liquefied petroleum gas, biogas, or electricity were classified as such, whereas those who reported using other fuels were classified as solid fuel users.

### Assessment of depression

The LASI study used the 10-item Center for Epidemiologic Studies Depression Scale (CES-D-10) to evaluate depressive symptoms, a short version of the CES-D-20, which is widely used for screening depression. It assesses the depressive symptoms of subjects with ten items about depressive feelings and behaviors in the past week, including three items, five items, and two items about the depressive, somatic, and positive effects, respectively (38). The score range of each item is 0–3 points, corresponding to “ <1 day,” “1–2 days,” “3–4 days,” and “5–7 days.” They were measured on a Likert scale. The scores of each item are summed, and the total score of the CES-D-10 ranges from 0 to 30. The CES-D-10 has good reliability and validity in older adults (39). According to a previous study, a CES-D-10 score of more than 10 was defined as a symptom of depression (40).

### Evaluation of cognition

The methods of evaluating cognition from the HRS were used in the present study, which includes several aspects of cognition, such as orientation, function, arithmetic, object naming, and word recall. In the LASI study, subjects were asked to perform word repetition, the orientation of time and place, backward counting, serial computation, implementation of paper folding and pentagon drawing, and object naming to measure their memory ability. During a session on word repetition, subjects immediately recited a list of words that had been shown to them before, and the procedure was to assess their memory function (score from 0 to 10). The test of the orientation of time and place made subjects identify the place, day, month, and year to measure the orientation score (from 0 to 4). The arithmetic test consisted of backward counting, serial seven, and computation, and the scores were 0–2, 0–5, and 0–2, respectively. In the implementation test, subjects were asked to fold a paper and draw scores from 0 to 3 and from 0 to 1. In addition, subjects were required to name and identify a specific object (score from 0 to 2). The scores of all domains were calculated into cognitive scores from 0 to 43 to assess older adults' cognition, and higher cognition scores indicated better cognitive health in older adults. Subjects with lower than 10% cognitive scores were identified as having cognitive impairment.

### Measurement of covariates

We included the following factors as covariates: The sociodemographic information included age (continuous), gender (male, female), education (never, middle school or under, secondary or above), marital status (married or in a relationship, widowed, or other), living area (urban or rural), whether to work (yes, no), economic status (tertile group-low/middle/high), caste (scheduled caste, scheduled tribe, other backward class, no or other castes), religion (Christian, Muslim, Hindu, or other); biological behavior information included moderate physical activity (more than once a week or hardly ever), body mass index (BMI) (<18.5, ≥18.5 and <25, ≥25 and <30, ≥30), drinking (never, current, ever), smoking (never, current, ever), combined chronic diseases (CCDs) (0, 1, 2, 3, or more), sleep disorder (no, yes); house environment information included other indoor pollution (no, yes), and indicators of poor housing quality (0, 1, 2, 3, 4, 5).

Several variables need to be explained in this study. Economic status was defined by annual per capita consumption expenditure. BMI (kg/m^2^) is defined as the weight (kg)/square of height (m). CCDs include hypertension, diabetes, tumors, lung disease, chronic heart disease, stroke, arthritis, mental disease, Alzheimer's disease, hypercholesterolemia, asthma, congestive heart failure, heart attack, abnormal heart rate, osteoporosis, abnormal thyroid function, digestive disease, skin disease, kidney stones, presbyopia, cataracts, glaucoma, myopia, hyperopia, tooth decay, and periodontal disease. Sleep disorder was identified as any of the following five situations that subjects reported: difficulty sleeping, waking up at night, waking up early, feeling sleepy during the day, and taking medicine to help sleep. Other indoor pollution included incense sticks (agarbatti), mosquito coils, liquid vaporizers/mosquito repellents/mats, fast cards/sticks/cakes, or if the housing respondent reports that a usual member of their household smokes inside their home. The subjects' answers (no, yes) to the above substances were analyzed. Indicators of poor housing quality were used to assess the quality of houses, which consisted of five indicators: house material, sanitary facilities, electric power, water source, and crowding (41). One score was recorded for each missing indicator of the subject's house, and the total score range was 0–5. The higher the score indicates, the worse the house quality.

### Statistical analysis

In the present study, we classified participants into two groups based on solid fuel use, and we presented categorical and continuous variables as percentages and means ± standard deviations. Multivariable logistic regression models evaluated the associations of solid fuel use with depressive symptoms and cognitive impairment. Multivariable linear regression models assessed the associations of solid fuel use with the CES-D-10 score and cognitive score. We used five models in each analysis: the unadjusted model, model I (adjusted for age, gender, and BMI), model II (adjusted for age, gender, and BMI, education, marriage, living area, whether someone works, caste, religion, and economic status), model III (adding indicators of drinking, smoking, sleep disorders, CCDs, and vigorous physical activity into model II), model IV (adding other indoor pollution and indicators of poor housing quality). Subgroup and interaction analyses were performed and stratified by gender, caste, living area, education, economic status, smoking, drinking, and BMI to explore potential effect modification. The sample size for each analysis is shown in the Supplementary material. The results with *p* < 0.05 and 95% confidence intervals (95% CI) were considered statistically significant. All analyses were performed by the statistical software package R 4.1.2 (http://www.R-project.org, The R Foundation).

## Result

### Characteristics of the study population

A total of 29,789 participants over 60 years old were included in this study. Of whom, there were 15,507 females and 14,282 males, and the total mean age was 68.7 years. For solid fuel, 14,203 participants reported using solid fuel at home, and 15,586 participants reported using clean fuel. Compared with those using clean fuel, solid fuel users showed a higher mean CES-D-10 score (solid fuel users: 10.2; clean fuel users: 9.5) and a higher rate of depression (solid fuel users: 49.8%; clean fuel users: 42.6%). The cognitive score of solid fuel users was 21.6, which was lower than that of clean fuel users (25.4). The incidence of cognitive impairment was 23.9% in solid fuel users, which was higher than that in clean fuel users (11.5%). Solid fuel users were more likely to have a low education level, live in the village, be unemployed, have low economic status, have a low BMI, drink alcohol, smoke, and live with other indoor pollution. However, there was no significant difference in age, gender, marriage status, or indoor pollution between clean and solid fuel users. Table 1 shows the characteristics of the study participants classified by using solid fuel.

**Table 1:** Characteristics of participants using solid fuel or using clean fuel.

**Characteristics**	**Total**	**Using clean fuel**	**Using solid fuel**	***P*** **-Value**
Numbers	29,789	15,586	14,203	
Age, year	68.7 ± 7.4	68.8 ± 4.2	68.6 ± 7.3	0.141
Gender, *n* (%)				0.397
Female	15,507 (52.1%)	8,050 (51.6%)	7,457 (52.5%)	
Male	14,282 (47.9%)	7,536 (48.4%)	6,746 (47.5%)	
CES-D-10 scores	9.83 ± 4.10	9.46 ± 4.17	10.23 ± 4.00	< 0.001
Depression using a cutoff of 10				< 0.001
< 10	16,074 (54.0%)	8,951 (57.4%)	7,123 (50.2%)	
≥10	13,715 (46.0%)	6,635 (42.6%)	7,080 (49.8%)	
Cognitive score	23.5 ± 7.1	25.4 ± 7.0	21.6 ± 6.7	< 0.001
Cognitive impairment				< 0.001
No	24,604 (82.6%)	13,794 (88.5%)	10,810 (76.1%)	
Yes	5,185 (17.4%)	1,792 (11.5%)	3,393 (23.9%)	
Education, *n* (%)	< 0.001			
Never	15,956 (53.6%)	6,379 (40.9%)	9,577 (67.4%)	
Middle school or under	9,303 (31.2%)	5,379 (34.5%)	3,924 (27.6%)	
Secondary or above	4,530(15.2%)	3,828 (24.6%)	702 (4.9%)	
Marriage status, *n* (%)				0.061
Married or partnered	19,195 (64.4%)	10,138 (65.0%)	9,057 (63.8%)	
Widowed	9,988 (33.5%)	5,143 (33.0%)	4,845 (34.1%)	
Others	606 (2.0%)	305 (2.0%)	301 (2.1%)	
Living area, *n* (%)				< 0.001
Urban	10,029 (33.7%)	8,493 (54.5%)	1,536 (10.8%)	
Rural	19,760 (66.3%)	7,093 (45.5%)	12,667 (89.2%)	
Whether work				< 0.001
No	19,472 (65.4%)	11,097 (71.2%)	8,375 (59.0%)	
Yes	10,317 (34.6%)	4,489 (28.8%)	5,828 (41.0%)	
Caste, *n* (%)				< 0.001
Scheduled caste	4,884 (16.5%)	2,118 (13.7%)	2,766 (19.6%)	
Scheduled trible	4,925 (16.6%)	1,431 (9.3%)	3,494 (24.7%)	
Other backward class	11,291 (38.1%)	6,130 (39.7%)	5,161 (36.5%)	
No or other caste	8,484 (28.7%)	5,774 (37.4%)	2,710 (19.2%)	
Religion, *n* (%)				< 0.001
Others	1,467 (4.9%)	906 (5.8%)	561 (4.0%)	
Hindu	21,846 (73.3%),	11,525 (73.9%)	10,321 (72.7%)	
Muslim	3,490 (11.7%)	1,819 (11.7%)	1,671 (11.8%)	
Christian	2,985 (10.0%)	1,336 (8.6%)	1,649 (11.6%)	
Economic status, *n* (%)				< 0.001
Low	10,560 (35.5%)	3,548 (22.8%)	7,012 (49.4%)	
Middle	10,039 (33.7%)	5,418 (34.8%)	4,621 (32.5%)	
High	9,188 (30.8%)	6,620 (42.5%)	2,568 (18.1%)	
BMI, *n* (%)				< 0.001
< 18.5	6,372 (23.3%)	1,961 (13.8%)	4,411 (33.5%)	
≥18.5, < 25	14,379 (52.6%)	7,325 (51.6%)	7,054 (53.6%)	
≥25, < 30	5,044 (18.4%)	3,644 (25.7%)	1,400 (10.6%)	
≥30	1,564 (5.7%)	1,264 (8.9%)	300 (2.3%)	
Drinking, *n* (%)				< 0.001
Never	24,647 (82.8%)	13,313 (85.5%)	11,334 (79.8%)	
Current	2,661 (9.0%)	1,133 (7.3%)	1,528 (10.8%)	
Ever	2,469 (8.3%)	1,131 (7.3%)	1,338 (9.4%)	
Smoking, *n* (%)				< 0.001
Never	23,734 (79.7%)	12,930 (83.0%)	10,804 (76.1%)	
Current	4,205 (14.1%)	1,714 (11.0%)	2,491 (17.5%)	
Ever	1,832 (6.2%)	928 (6.0%)	904 (6.4%)	
Vigorous physical activity, *n* (%)				< 0.001
More than or equal to once a week	7,979 (26.8%)	3,508 (22.5%)	4,471 (31.5%)	
Hardly ever	21,791 (73.2%)	12,070 (77.5%)	9,721 (68.5%)	
CCDs				< 0.001
0	4,987 (16.9%)	1,665 (10.8%)	3,322 (23.6%)	< 0.001
1	6,312 (21.4%)	2,795 (18.1%)	3,517 (25.0%)	
2	6,073 (20.6%)	3,221 (20.8%)	2,852 (20.3%)	
3, or more than 3	12,169 (41.2%)	7,785 (50.3%)	4,384 (31.1%)	
Sleep disorder				
Without sleep disorder	25,153 (84.5%)	13,231 (84.9%)	11,922 (83.9%)	< 0.001
With sleep disorder	4,627 (15.5%)	2,347 (15.1%)	2,280 (16.1%)	
Other indoor pollution, *n* (%)				< 0.001
No	3,755 (12.6%)	1,616 (10.4%)	2,139 (15.1%)	
Yes	26,031 (87.4%)	13,967 (89.6%)	12,064 (84.9%)	
Indicators of poor housing quality, *n* (%)				< 0.001
0	11,265 (37.8%)	8,525 (54.7%)	2,740 (19.3%)	
1	9,620 (32.3%)	4,601 (29.5%)	5,019 (35.3%)	
2	5,489 (18.4%)	1,789 (11.5%)	3,700 (26.1%)	
3	2,712 (9.1%)	608 (3.9%)	2,104 (14.8%)	
4	696 (2.3%)	63 (0.4%)	633 (4.5%)	
5	7 (< 0.1%)	0 (0.0%)	7 (< 0.1%)	

SD, standard deviation; BMI, body mass index.

Mean ± SD for continuous variables: The P-value was calculated by the weighted linear regression model.

Number (%) for Categorical variables: The P-value was calculated by weighted chi-square test.

### Association of solid fuel use at home with depression and cognitive impairment

We found a positive and significant association between solid fuel use and depression in logistic regression analysis. In the unadjusted model, using solid fuel was associated with a higher risk of depression, with an OR of 1.37. In model I, adjusted for age, gender, and BMI, the relationship between solid fuel use and depression remained unchanged (OR 1.27, 95% CI 1.21–1.33). In model II, the OR of depression from solid fuel use decreased after adjusting for sociodemographic information, which still met statistical significance (OR 1.15, 95% CI 1.08–1.22). After adding covariates of biological information, solid fuel use was significantly correlated with depression (OR 1.18, 95% CI 1.11–1.25) in model III. In the fully adjusted model (model IV), the significance of the relation between solid fuel use and depression decreased, with an OR of 1.09 (95% CI 1.03–1.16). There was a trend for the OR of depression from solid fuel consumption to decrease when additional covariates were included in the analysis.

With the similarity of trend, there was a significant association between solid fuel use and cognitive impairment in all models based on the logistic regression analysis that domestic solid cooking fuel use was associated with a higher prevalence of cognitive impairment (unadjusted model, OR 2.42, 95% CI 2.27–2.57; mode l I, OR 2.14, 95% CI 1.99–2.30; model II, OR 1.29, 95% CI 1.18–1.40; model III, OR 1.27,95% CI 1.16–1.38; model IV, OR 1.21, 95% CI 1.11–1.32).

We considered the CES-D-10 score a continuous variable in linear regression analysis, and the results were similar to those from logistic regression analysis. In the unadjusted model, solid fuel users had a higher CES-D-10 score than clean fuel users (β 0.77, 95% CI 0.67–0.86). In mode I, those using solid fuel were associated with a 0.60 higher CES-D-10 score than clean fuel users. The result stating solid fuel use was related to a higher CES-D-10 score remained unchanged in model II (β 0.38, 95% CI 0.27–0.49). After adjusting for biological information, the CES-D-10 score of solid fuel users was 0.39 points higher than that of clean fuel users (95% CI, 0.27–0.50). In the fully adjusted model, the difference between solid and clean fuel users decreased but met significance (β 0.23, 95% CI 0.12–0.35). Solid fuel use was negatively related to cognitive scores. Compared with those who used clean fuel, the cognitive score decreased by 3.89 in those who used solid fuel in the unadjusted model (95% CI, −3.96 to −3.65). With more covariates included in analyses, the OR-value of the cognitive score between solid fuel users and clean fuel users decreased. In the fully adjusted model, the relation between solid fuel use and the cognitive score remained unchanged (model I, β −2.91, 95% CI −3.07 to −2.76; mode lII, β −0.78,95% CI −0.93 to −0.62; model III, β −0.75, 95% CI −0.91 to −0.60; model IV, β −0.63, 95% CI −0.79 to −0.47). Table 2 presented the association between solid fuel use and depression and cognitive impairment by logistic regression and linear regression analysis.

**Table 2:** Association of using solid fuel with symptoms of depression, CES-D-10 score, cognitive impairment, and cognitive scores.

	OR/β (95% CI)				
Score of CES-D-10	Unadjusted model	mode I	model II	model III	model IV
Categorical variable (depression)^*^					
Using clean fuel	Reference	Reference	Reference	Reference	Reference
Using solid fuel	1.37 (1.28, 1.40)	1.27 (1.21, 1.33)	1.15 (1.08, 1.22)	1.18 (1.11, 1.25)	1.09 (1.03, 1.16)
Continuous variable					
Using clean fuel	Reference	Reference	Reference	Reference	Reference
Using solid fuel	0.77 (0.67, 0.86)	0.60 (0.50, 0.70)	0.38 (0.27, 0.49)	0.39 (0.27, 0.50) < 0.0001	0.23 (0.12, 0.35)
Cognitive score	Unadjusted model	mode I	model II	model III	model IV
Categorical variable (cognitive impairment)^**^					
Using clean fuel	Reference	Reference	Reference	Reference	Reference
Using solid fuel	2.42 (2.27, 2.57)	2.14 (1.99, 2.30)	1.29 (1.18, 1.40)	1.27 (1.16, 1.38)	1.21 (1.11, 1.32)
Continuous variable					
Using clean fuel	Reference	Reference	Reference	Reference	Reference
Using solid fuel	−3.80 (−3.96, −3.65)	−2.91 (−3.07, −2.76)	−0.78 (−0.93, −0.62)	−0.75 (−0.91, −0.60)	−0.63 (−0.79, −0.47)

CES-D-10, the Center for epidemiologic studies depression scale; OR, odds ratio; β, effect sizes; 95% CI, 95% confidence interval.

* Depression was defined as CES-D-10 score over 10;

** Cognitive impairment was identified as a cognitive score lower than 10 %; Model I: adjusted for age, gender, and BMI; Model II: adjusted for age, gender, BMI, education, marriage, live area, whether works, caste, religion, and economic status; Model III: adjusted for age, gender, BMI, education, marriage, live area, whether works, caste, religion, economic status, drinking, smoking, sleep disorder, CCDs, and vigorous physical activity; Model IV: adjusted for age, gender, BMI, education, marriage, live area, whether works, caste; religion, economic status, drinking, smoking, sleep disorder, CCDs, vigorous physical activity, other indoor pollution, and indicators of poor housing quality.

### Subgroup analyses and interaction analyses

In this present study, subgroup analyses were stratified by gender (female, male), education (never, middle school or under, and secondary or above), living area (urban, rural), caste (scheduled caste, scheduled tribe, other backward class, no or other castes), and economic status (tertile group), BMI (< 18.5, ≥18.5 and <25, ≥25 and <30, ≥30), smoking (never, current, ever), and drinking (never, current, ever). We observed significant interactions in the association between solid fuel use and depression on the relation of solid fuel with depression, in different gender and smoking (p for interaction: gender 0.009, smoking 0.002). After classifying participants by gender, the association between solid fuel use and depression still existed among females (OR 1.18, 95% CI 1.08–1.28), while the relationship between the two did not have statistical differences in males (OR 1.01, 95% CI 0.92–1.10). Those who never smoked were more likely to suffer from depression due to solid fuel use (OR 1.14, 95% CI 1.07–1.23). The correlation of solid fuel use with depression disappeared among those who ever smoked or currently smoked. We did not observe a significant interaction of BMI, economic status, caste, living area, education, or drinking on the association between solid fuel use and depression.

**Figure 2:**
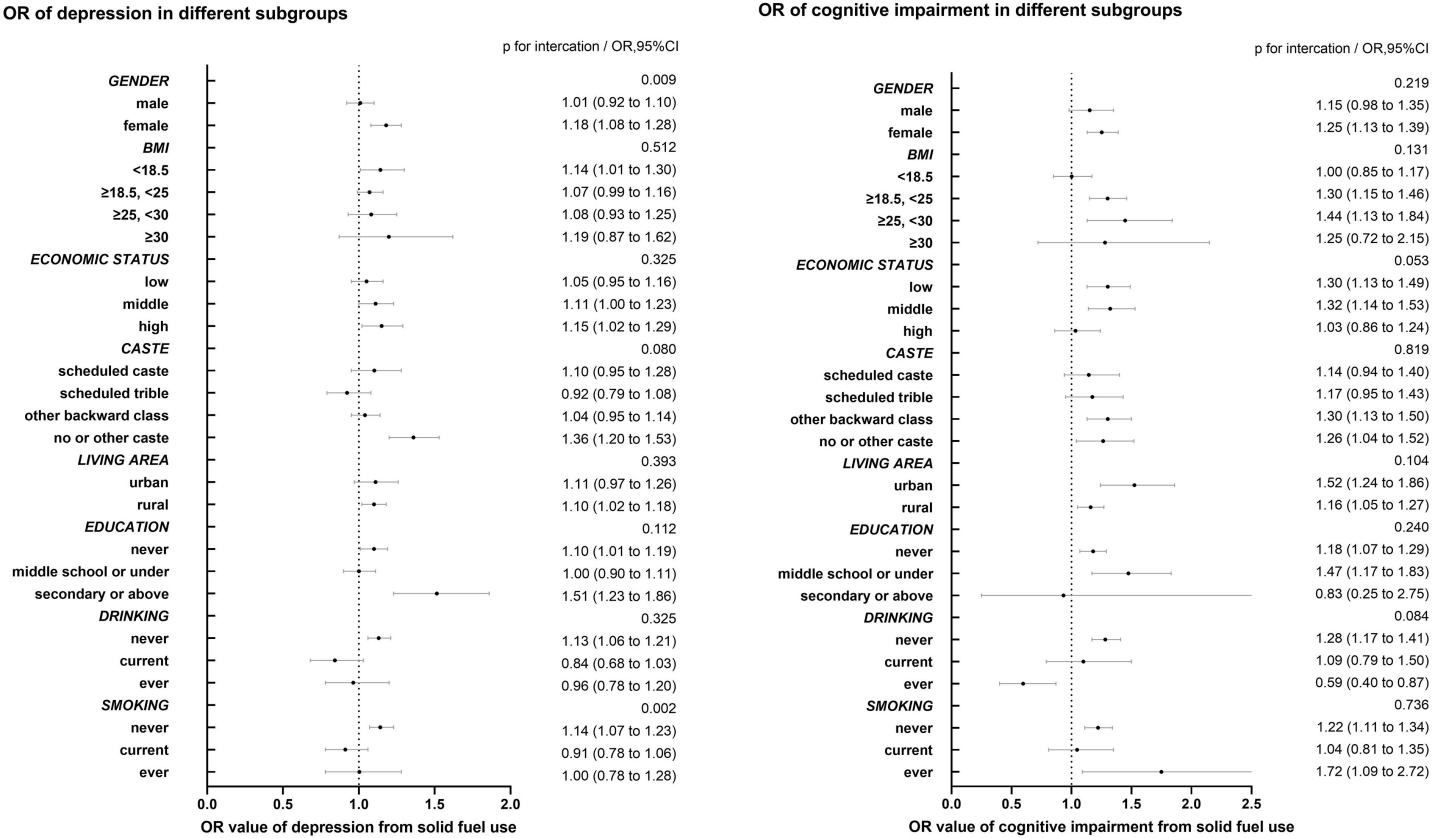
Subgroup analysis in the association of solid fuel use with depression and cognitive impairment. Subgroup analysis were adjusted by age, gender, BMI, education, marriage, live age, whether works, caste, religion, economic status, drinking, smoking, sleep disorder, CCDs, vigorous physical activity, other indoor pollution, indicators of poor housing quality.

After adjusting for all confounders, no significant interaction of gender, education, living area, caste, BMI, smoking, or drinking on the correlation between solid fuel use and cognitive impairment was observed. We did not obtain any evidence to prove the existence of differences in the relation of solid fuel use with cognitive impairment in different subgroups. Subgroup analysis is presented in Figure 2.

## Discussion

This cross-sectional study assessed the associations of solid fuel use with depression and cognitive impairment. Solid fuel use was associated with a 37% higher prevalence of depression and a 142% higher prevalence of cognitive impairment than clean fuel use in the unadjusted model. After adjusting biological and social demographic covariates, the association of solid fuel use with depression and cognitive impairment remained unchanged. After adding house quality and indoor pollution as covariates into the analysis, the value of solid fuel use on depression and cognitive impairment decreased. However, the results still showed a significant difference. In the subgroup analysis, we found that females and non-smokers suffered a higher prevalence of depression related to solid fuel use. The relationship between solid fuel and cognitive impairment was stable in different subgroups.

We analyzed the association of solid fuel use with depression and cognitive impairment among older adults based on a population sample from representable LMICs. At the same time, the current study included the largest and latest sample size among similar studies (29,789). The prevalence of depression and cognitive impairment in LMICs is increasing, especially in the elderly population, but the relevant research has been insufficient in recent years. For the extensive research on the adverse health effects of air pollution, reducing the use of solid fuels has been considered a way to improve indoor pollution (42, 43). Previous studies have also demonstrated the enormous economic burden of indoor solid fuel use (44). Our research shows that the adverse effects on health caused by solid fuels still exist based on the latest data from LASI Wave 1, even though such negative consequences had been reported beforehand. As noted previously, in the different subgroups of gender, education, BMI, smoking, or drinking, there are different susceptibilities to depression, so it is necessary to determine the association of solid fuel use with depression and cognitive impairment in those subgroups (45–54). In India, populations with different economic statuses, living areas, and castes used different ratios of solid fuel. According to our findings, subgroup analyses based on economic status, living area, and castes used different ratios of solid fuel, according to our findings of subgroup analyses.

The associations between the use of solid fuel and depression and cognitive impairment have been provided by previous studies, but the underlying molecular mechanism for the effects of air pollution on depression and cognitive impairment is not clear (30, 36, 55–60). Several explanations show possible links. First, solid fuel increases oxidative stress (OS) in the human body through a high concentration of PM and chemical substances, which promotes the progression of depression and cognitive impairment. OS plays a major role in neurodegeneration, which involves depressive pathogenesis (61, 62). Animal experiments have shown that PM_2.5_ promotes oxidative stress through the Nrf2/NF-κB pathway and increases the level of inflammatory cytokines (63). PM was also related to the activation of the nuclear transcription factors Nrf-2 and NF-κB in male rats, and PM may lead to physiological changes in the central nervous system (64). A study on females with solid fuel use suggested that solid fuel users had 32% more leukocytes in circulation, and reactive oxygen species (ROS) were generated at higher levels in neutrophils, lymphocytes, eosinophils, and alveolar macrophages (65). Second, metabolic alterations resulting from PM and chemical substances may lead to depression. After short- and long-term exposure to PM, triglyceride levels increased; after long-term exposure to PM, free fatty acid levels also increased (66). PM also hurts glucose metabolism: exposure to PM_2.5_ increases insulin resistance (67). PAHs, NO_2_, and CO are related to biological toxicity (68–70). Similarly, higher levels of triglycerides and free fatty acids were observed among those living with depression, and it was reported that higher glucose concentrations of the pregenual anterior cingulate were associated with major depressive disorder (71–73). In a study from China, hyperlipidemia and hyperglycemia were related to cognitive impairment (53). Finally, solid fuel use was related to depression and cognitive impairment, which may be linked to chronic disease. Solid fuel users suffer a higher risk of chronic disease. A meta-analysis suggested that multimorbidity was related to depression and cognitive impairment, which means that chronic diseases resulting from solid fuel led to the emergence of depression and cognitive impairment (26, 53, 74, 75).

As reported by previous studies, using solid fuel was related to depression, which is consistent with the results of our study (36, 55–59). The adjusted OR and 95% CI of solid fuel use for depression were OR 1.09 and 95% CI 1.03–1.16 in our study, respectively. The value differed from previous studies due to the difference in study populations and definitions of solid fuel use and depression. In the subgroup analysis, we found that females and non-smokers suffer a higher OR from solid fuel use for depression, and there was no interaction on economic status, which was different from the findings in previous studies. Contrary to our findings, research from China found no connection between gender and smoking solid fuel and depression (59). The India Human Development Survey study found that in 98% of households, females cooked, which seems to explain why females have a higher OR of solid fuel use for depression than males in the Indian population due to the higher exposure to pollutants from solid fuel (34). Secondhand smoke exposure is related to depressive symptoms among those who never smoke (76). People who are not exposed to tobacco appear to be more sensitive to air pollution, and nonsmokers who suffer a higher OR from solid fuel use for depression need further study. In a previous study, associations between using solid fuel and depression were generally higher in females and those with low household economic levels among older Chinese adults (55). In our study, low household economic levels were not related to a higher risk of depression, which may be associated with the broader use of solid fuel in India. In the subgroup of caste, different relations of solid fuel to depression were observed for the differences in health and lifestyle (77).

The association between solid fuel use and cognitive impairment has been frequently determined in China, while the evidence from other areas is limited (30, 60, 78). A study from Mexico suggested that solid cooking fuels may represent a risk factor for cognitive decline (79). Additionally, the association between solid fuel and depressive symptoms for ten years depended on the WHO Study on Global AGEing and Adult Health (SAGE) (80). A meta-analysis suggested that a 24% higher risk of depression was related to solid fuel use, while the OR of solid fuel use for depression was 1.08, according to our findings (81). Compared with another study based on the LASI study, we excluded those younger than 60 and involved indicators of poor housing quality as covariants to accurately evaluate the relationship between solid fuel use and cognitive impairment among older adults (82). Evidence from a study from northern China suggested that domestic solid fuel consumption was a dose-dependent risk factor for cognitive impairment (83). Although we did not find a significant p for interaction in subgroups between solid fuel use and cognitive impairment, there were still subgroups suffering from the different effects of solid fuel on cognitive impairment. Among males, we found a negative relationship between solid fuel and cognitive impairment compared with females because of their lower exposure to pollutants (34). There was no significant association between solid fuel use and cognitive impairment in subgroups of high economic status and high educational status, which indicates the protective effect of the two (51, 84). In our study, the correlations between solid fuel use and cognitive impairment were stable and consistent with the findings from a previous study (82).

There were several limitations to this study. First, the present study is cross-sectional, making it difficult to identify the causal relationship between solid fuel use and depression or cognitive impairment. Second, our study's definitions of depression and cognitive impairment were based on screening tools (the CES-D-10 and HRS) but not diagnostic criteria. The CES-D-10 was primarily used for assessing depression symptom severity (40). Although the HRS cognitive assessment method is widely used in various studies as a face-to-face interview-based test, there are still some limitations (85). Third, the patient's answer determined whether the subjects used solid fuel, which may not have been objectively observed and have certain recall biases. Fourth, due to the lack of relevant data, we did not evaluate the impact of the total time of solid fuel use every day and the total time of solid fuel use on the association between solid fuel use and depressive symptoms. Fifth, because of the lack of kitchen ventilation, we used indicators of poor housing quality to assess the quality of the house. Indicators of poor housing quality helped us reduce the statistical bias caused by the lack of kitchen ventilation information in a previous study (41). Sixth, due to the low concentration of air pollution to which the subjects were exposed, we did not analyze the dose relationship of air pollutant concentrations with depression and cognitive impairment. Seventh, the population we studied was comprised of elderly individuals over the age of 60 and did not include people under 60. The relationship of solid fuel use with depression and cognitive impairment in these populations needs further verification.

Although evidence from a 10-year study suggested a link between solid fuel use and depression, solid fuel use was also related to depression in our research, indicating that solid fuel's effect still existed. Therefore, more government measures to improve indoor pollution are needed (59, 80). Older adults should use clean fuel at home for cooking, which is beneficial for their mental health and cognition. Our study first provided the association of solid fuel use with depression and depression at the same time among older adults, and more policies on reducing the use of solid fuels, promoting clean fuels, and improving air quality are needed.

## Conclusion

Domestic solid cooking fuel use was associated with the increased prevalence of depression and cognitive impairment among older adults in India.

## Data availability statement

The datasets presented in this study can be found in online repositories. The names of the repository/repositories and accession number(s) can be found at: https://doi.org/10.25549/h-lasi.

## Ethics statement

The studies involving human participants were reviewed and approved by the Indian Council of Medical Research. The patients/participants provided their written informed consent to participate in this study.

## Author contributions

SQ, XZ, and QW were involved in the conception and design of the work. YJ, LD, and XX contributed to the acquisition, analysis, and interpretation of the data. YJ and YL finished the first draft. SQ and BD revised the manuscript for important intellectual content. All authors reviewed and approved the final version.

## Funding

This work was supported by programs from the Science and Technology Department of Sichuan Province (2021YJ0462), the 1.3.5 Project for Disciplines of Excellence at West China Hospital, Sichuan University (ZYGD20010), the Health and Family Planning Commission of Sichuan Province (20PJ039), the China Postdoctoral Science Foundation (2020M670057ZX), a postdoctoral research project at West China Hospital, Sichuan University (2019HXBH092), and the Chengdu Science and Technology Bureau Major Science and Technology Application Demonstration Project (2019YF0900083SN).

## Conflict of interest

The authors declare that the research was conducted in the absence of any commercial or financial relationships that could be construed as a potential conflict of interest.

## Publisher's note

All claims expressed in this article are solely those of the authors and do not necessarily represent those of their affiliated organizations, or those of the publisher, the editors and the reviewers. Any product that may be evaluated in this article, or claim that may be made by its manufacturer, is not guaranteed or endorsed by the publisher.
