# Supplementary material for: Association between the domestic use of solid cooking fuel and increased prevalence of depression and cognitive impairment in a big developing country: A large-scale population-based study
Source: Front Public Health. 2022 Nov 24;10:1038573. doi: 10.3389/fpubh.2022.1038573 (PMC9731231; doi:10.3389/fpubh.2022.1038573)
Supplement: Supplementary file 1 [file Data_Sheet_1.docx]

Supplementary Material

# Supplementary table

Supplementary table 1: Sample size of each model of logistic regression and linear regression

| Outcome | Exposure | Non-adjusted | Adjust I | Adjust II | Adjust Ⅲ | Adjust Ⅳ |
| --- | --- | --- | --- | --- | --- | --- |
| CED10 | solid fuel | 29789 | 27359 | 27172 | 26916 | 26916 |
| Depression 4-10 | solid fuel | 29789 | 27359 | 27172 | 26916 | 26916 |
| COGNITIVE.SCORE | solid fuel | 29789 | 27359 | 27172 | 26916 | 26916 |
| COGNITIVE.IMPIREMENT | solid fuel | 29789 | 27359 | 27172 | 26916 | 26916 |

Supplementary table 2: Sample size of each subgroup in subgroup analysis

| Sub-group | Depression | Cognitive impairment |
| --- | --- | --- |
| Gender |  |  |
| Male | 14282 | 14282 |
| Female | 15507 | 15507 |
|  |  |  |
| BMI |  |  |
| <18.5 | 6372 | 6372 |
| ≥18.5, <25 | 14379 | 14379 |
| ≥25, <30 | 5044 | 5044 |
| ≥30 | 1564 | 1564 |
|  |  |  |
| Economic status |  |  |
| Low | 10560 | 10560 |
| Middle | 10039 | 10039 |
| High | 9188 | 9188 |
|  |  |  |
| Caste |  |  |
| scheduled caste | 4884 | 4884 |
| scheduled trible | 4925 | 4925 |
| other backward class | 11291 | 11291 |
| no or other caste | 8484 | 8484 |
|  |  |  |
| Living area |  |  |
| urban | 10029 | 10029 |
| rural | 19760 | 19760 |
|  |  |  |
| Education |  |  |
| never | 15956 | 15956 |
| middle school or under | 9303 | 9303 |
| secondary and higher secondary | 4530 | 4530 |
|  |  |  |
| Drinking |  |  |
| Never | 24647 | 24647 |
| Current | 2661 | 2661 |
| Ever | 2469 | 2469 |
|  |  |  |
| Smoking |  |  |
| Never | 23734 | 23734 |
| Current | 4205 | 4205 |
| Ever | 1832 | 1832 |
